# The combined treatment with novel platinum(II) complex and anti-MUC1 increases apoptotic response in MDA-MB-231 breast cancer cells

**DOI:** 10.1007/s11010-015-2486-z

**Published:** 2015-06-27

**Authors:** Agnieszka Gornowicz, Anna Bielawska, Robert Czarnomysy, Halina Gabryel-Porowska, Anna Muszyńska, Krzysztof Bielawski

**Affiliations:** Department of Biotechnology, Medical University of Bialystok, Kilinskiego 1, 15-089 Białystok, Poland; Department of Synthesis and Technology of Drugs, Medical University of Bialystok, Kilinskiego 1, 15-089 Białystok, Poland; Deparment of Medical Chemistry, Medical University of Bialystok, Kilinskiego 1, 15-089 Białystok, Poland

**Keywords:** Anti-MUC1, Anticancer drugs, Platinum complexes, Targeted therapy, Apoptotic markers

## Abstract

New strategy of cancer’s targeting treatment is combining monoclonal antibodies with chemotherapeutic agents. An important goal of targeted therapy appears to be a transmembrane glycoprotein type I—mucin 1 (MUC1), which is overexpressed in tumors of epithelial origin, especially in breast cancer. The goal of the study was to check the effect of monoclonal antibody against MUC1 with novel platinum(II) complex (Pt12) on selected aspects of apoptosis in human MDA-MB-231 breast cancer cells. The number of apoptotic and necrotic cells was measured using annexin V binding assay. The decrease of mitochondrial membrane potential (MMP) and DNA fragmentation was analyzed. Finally, the influence of novel platinum(II) complex (Pt12) used with anti-MUC1 on the concentration of selected markers of apoptosis such as Bax, caspase-8, -9, and caspase-3 was performed using ELISA. The results from combined treatment were compared with those obtained using monotherapy. In our study, we proved that anti-MUC1 used in combination with Pt12 strongly induced apoptosis in MDA-MB-231 breast cancer cell line. The effect was stronger than treatment with Pt12, cisplatin, anti-MUC1, and anti-MUC1 used with cisplatin. We also observed the highest decrease of MMP and the strongest DNA fragmentation after such a combined treatment. The results obtained from ELISA showed increased concentration of Bax, caspases-8, -9, -3 compared to monotherapy. Our study proved that Pt12 together with anti-MUC1 strongly induced apoptosis in estrogen-negative breast cancer cell line (MDA-MB-231). The apoptosis may go through extrinsic pathway associated with caspase-8 as well as intrinsic pathway connected with caspase-9.

## Introduction

Currently, only 20–75 % of patients with cancer (melanoma 4–12 %) correspond to the standard treatment. The specific targeted therapy is one of the greatest achievements of medicine in the last decade. New strategy of cancer’s targeting treatment is combining monoclonal antibodies with chemotherapeutic agents. An important goal of targeting therapy appears to be a transmembrane glycoprotein type I—mucin 1 (MUC1) which is overexpressed in tumors of epithelial origin, especially in breast cancer [1–3]. In many tumor types, MUC1 expression correlates with aggressive, metastatic disease, poor response to therapy, and poor survival [4]. MUC1 was ranked by the National Cancer Institute as the second most promising candidate tumor antigen with a high clinical potential [5, 6]. MUC1 is also involved in the phenomenon of drug resistance and in the inhibition of induction of apoptosis. Increased expression of MUC1 induces thyroid cancer cell resistance to cisplatin, docetaxel and doxorubicin [7] and it induces the resistance to trastuzumab in breast cancer cells [8]. MUC1 inhibits the release of apoptotic markers and activation of caspase-3 in colon cancer cells treated with cisplatin [9]. MUC1 cytoplasmic domain inhibits the intrinsic apoptotic pathway by regulation of various signaling pathways associated with p53, FOXO3a, c-Abl, IkB complex, FADD, or Bax. Mucin domain that interacts with the pro-apoptotic protein Bax in the cytoplasm and mitochondria blocks its dimerization and leads to the inhibition of the releasement of cytochrome *c* [10]. The cytoplasmic domain of MUC1 may communicate with caspase-8 and Fas receptor thereby blocking activation of extrinsic apoptosis pathway [11]. A variable number tandem repeat (VNTR) domain of MUC1 is immunogenic, which led to the establishment of monoclonal antibodies which react with the epitopes of the domain. Monoclonal antibodies are used in the treatment of many diseases such as osteoporosis, asthma, and hematological malignancies: Hodgkin’s lymphoma, chronic lymphocytic leukemia, or solid tumors [12]. The mechanism of action of monoclonal antibodies that are used in tumor therapy is based on the destruction of cancer cells by inhibiting intracellular signal transduction which induces the process of cell death. This process is performed by the binding with tumor’s cells receptor and modulation of the receptor, or interfering with ligand binding and/or dimerization of the receptor [13]. Monoclonal antibodies may also stimulate the host immune system through antibody-dependent cell-mediated cytotoxicity (ADCC) or a complement-dependent cytotoxicity (CDC) [14].

In recent years, in our laboratory we obtained a series of novel amidine analogues of melphalan or chlorambucil [15–17] and dinuclear platinum complexes with high antitumor activity in human breast cancer cells [18, 19]. Our preliminary studies where we checked the combined effects of a monoclonal antibody against MUC1 used together with a novel dinuclear platinum complex (Pt12) showed high anti-proliferative properties and a strong cytotoxic activity in two breast cancer cell lines (MCF-7 and MDA-MB-231). This effect was much stronger than the treatment with anti-MUC1, cisplatin, Pt12, or cisplatin in the presence of anti-MUC1 [20]. A key element of the research is to understand the molecular mechanisms of apoptosis induced by anti-MUC1 in combination with the new platinum complex in MDA-MB-231 cells. The breast cancer cell line (MDA-MB-231) is characterized by intermediate response to chemotherapy compared to MCF-7, which is often chemotherapy responsive [21]. The purpose of the study was to check the influence of monoclonal antibody against MUC1 on sensitivity of MDA-MB-231 cells to chemotherapeutic agents (Pt12, cisplatin). We decided to analyze the effect of such a treatment on selected aspects of apoptosis: number of early and late apoptotic cells, mitochondrial membrane potential (MMP), DNA fragmentation, and concentration of apoptotic markers (Bax, caspases-3, -8, -9).

## Materials and methods

### Materials

Dimethylformamide, K_2_PtCl_4_, KI, acetone, 4-ethylpyridine, diethyl ether, methanol, cisplatin, monoclonal antibody anti-MUC1 GP1.4 were purchased from Sigma Chemical Co. (USA). Stock cultures of human MDA-MB-231 breast cancer cells were purchased from the American Type Culture Collection (USA). Dulbecco’s minimal essential medium (DMEM) and fetal bovine serum (FBS) used in a cell culture were products of Gibco (USA). Glutamine, penicillin, and streptomycin were obtained from Quality Biologicals Inc. (USA). FITC Annexin V Apoptosis Detection Kit II, JC-1 MitoScreen Kit, Apo-Direct Kit were a product of BD Pharmigen. ELISA’s kits were purchased from Uscn Life Science Inc. and BioVendor. The chemical synthesis and structure of Pt12 was presented in publication [20].

### Cell culture MDA-MB-231

Estrogen-negative breast cancer MDA-MB-231 cells were maintained in DMEM (Dulbecco’s Minimal Essential Medium) supplemented with 10 % FBS, 2 mM glutamine, 50 U/mL penicillin, 50 mg/mL streptomycin at 37 °C in a humidified atmosphere containing 5 % CO_2_. Cells were incubated with anti-MUC1 (10 μg/mL), Pt12 (10 μM), Pt12 + anti-MUC1 (10 μM + 10 μg/mL), cisplatin (10 μM), cisplatin + anti-MUC1 (10 μM + 10 μg/mL) for 24 and 48 h and used for further experiments such as annexin V binding assay, determination of MMP and DNA fragmentation. MDA-MB-231 cells after 24 h of incubation with compounds in the same concentrations were used to prepare cell lysates. Briefly, trypsinized cells were washed three times with cold PBS and centrifuged at 1000×*g* for 5 min at 4 °C. The cells (1 × 10^6^) were suspended in lysis buffer for whole cell lysates. After centrifugation the supernatants were frozen immediately at −70°. The concentration of pro-apoptotic markers was measured. Cells without addition of compounds were treated as controls.

### Annexin V binding assay

Apoptosis was determined assessing phosphatidylserine exposure by Annexin V-FITC binding by means of the FITC Annexin V Apoptosis Detection Kit II according to the manufacturer’s instruction. Cells (10,000 cell measured) were analyzed in a flow cytometer (BD FACSCanto II flow cytometer, CA, USA). Annexin V bounds with high affinity to phosphatidylserine, and thus could be used to identify cells in all stages of the programmed cell death [22]. Propidium iodide (PI) exclusively stained cells with a disrupted cell membrane and could be used to identify late apoptotic and dead cells. Cells cultured in a drug-free medium were used as controls. Optimal parameter settings were found using a positive control (cells incubated with 3 % formaldehyde in buffer during 30 min on ice). Forward scatter (FS) and side scatter (SC) signals were detected on a logarithmic scale histogram. FITC was detected in the FL1 channel (FL1 539; threshold-value 52). Analysis was performed using the BD FACSCanto II flow cytometer, and results were analyzed with FACSDiva software (both from BD Biosciences Systems, San Jose, CA, USA). Dots with Annexin V−/PI−(Q3), Annexin V+/PI−(Q4), and Annexin V+/PI+(Q2) features represent intact, early apoptotic, and necrotic cells, respectively. The percent of apoptotic cells (early and late) were measured as: percentage of cells from the upper right square (color red) + percentage of cells from the lower right square (color blue).

### Determination of mitochondrial membrane potential

Disruption of the MMP was assessed using the lipophilic cationic probe 5,5,6,6-tetrachloro-1,1,3,3-tetraethylbenzimidazolcarbocyanine iodide (JC-1 MitoScreen kit; BD Biosciences) as described previously [23]. Briefly, unfixed cells were washed and resuspended in PBS supplemented with JC-1. Cells were then incubated for 15 min at room temperature (RT) in the dark, washed, and resuspended in PBS for immediate BD FACSCanto II flow cytometry analysis. The percentage of cells with disrupted MMP was calculated in the FACSDiva software (both from BD Biosciences Systems, San Jose, CA, USA).

### DNA fragmentation assay

DNA fragmentation was examined by the terminal deoxynucleotidyltransferase (TdT)-mediated dUTP nick end labeling (TUNEL) method using a commercial assay kit (APO-Direct Kit; BD Pharmingen, San Diego, CA, USA). After treatment, cells were fixed with 1 % paraformaldehyde in PBS (4 °C, 30 min), washed in PBS, and permeabilized with ice cold 70 % ethanol. The Apo-Direct Kit-TUNEL assay was performed as described by the manufacturer. Cells were analyzed in a BD FACSCanto II flow cytometer. In total, 10,000 events were collected per test sample. The results were analyzed in FACSDiva software (both from BD Biosciences Systems, San Jose, CA, USA). The percentage of TUNEL negative and positive cells was presented.

### Determination of pro-apoptotic Bax protein

The high sensitivity assay kit (Uscn Life Sci Inc.) was used to determine the concentration of pro-apoptotic Bax protein in cell lysates. The microtiter plate provided has been pre-coated with a monoclonal antibody specific to Bax. Standards and samples were added to the appropriate microtiter plate wells and incubated for 2 h at 37 °C. After first incubation step a biotin-conjugated polyclonal antibody specific for Bax was pipetted and incubated for 1 h at 37 °C. After washing away any unbound substances, avidin conjugated to horseradish peroxidase was added to each microplate well and incubated. After another aspiration and washing step a TMB substrate solution was added to each well. The enzyme-substrate reaction was terminated by the addition of a sulfuric acid solution and the color change was measured at a wavelength of 450 nm. The assay was performed in duplicate and the concentration of Bax in the samples was then determined by comparing the O.D. of the samples to the standard curve. Range of the standard curve for Bax was 0.78–50 ng/mL. The minimum detectable dose of human Bax was generally less than 0.32 ng/mL.

### Determination of caspase-8 and caspase-9

Caspase-8 and caspase-9 concentrations in cell lysates were determined using an enzyme-linked immunosorbent assay kit (BioVendor). A monoclonal antibodies specific for caspase-8 or caspase-9 have been pre-coated onto a microplate. Standards and samples (100 μL each) were pipetted into the wells in duplicate and antigen was bound by the immobilized antibody. The working solution of antibody was also added to all wells. Then the microplate was incubated for 2 h at RT. After washing away any unbound substances, an enzyme-linked polyclonal antibody specific for caspase-8 or caspase-9 (100 μL) was added to each well for 1 h at RT. Following a wash to remove any unbound antibody enzyme-reagent, a substrate solution (100 μL) was added to the wells for 15 min; the color developed in proportion to the amount of antigen bound in the initial step. Color development was stopped by phosphoric acid, and the intensity of the color was measured at a wavelength of 450 nm. The minimum detectable dose (MDD) of caspase-8 was 0.1 ng/mL and caspase-9 was: 0.4 ng/mL. The concentrations of the samples were calculated from the standard curve and ranged from 0.16 to 10 ng/mL for caspase-8 and 1.6–100 ng/mL for caspase-9. The results were presented in nanogram per milliliter (ng/mL). There was no cross-reactivity with other caspases.

### Determination of active caspase-3

The concentration of caspase-3 was checked using assay kit from Uscn Life Sci Inc. Samples and standards (100 μL/well) were added into wells and microplate was incubated for 2 h at 37 °C. After 2 h antibody (1:100) was added and plate was incubated for next 60 min at 37 °C. The washing procedure was performed and polyclonal antibody with enzyme was pipetted and kept for 30 min at 37 °C. After washing away any unbound substances substrate solution (90 μL/well) was added. The reaction was stopped by the addition of sulfuric acid. The absorbance was checked at the wavelength 450 nm. The concentration of caspase-3 was calculated from standard curve (0.156–10 ng/mL). The minimum detectable dose was 0.054 ng/mL. The results were presented in nanogram per milliliter (ng/mL).

### Statistical analysis

Experimental data were represented as mean ± SD since each experiment was repeated three times. One-way Anova, Dunnett’s multiple comparisons test was performed to demonstrate the difference between control (untreated cells) and the different treatments. A statistically significant difference was defined at *p* < 0.05. Statistical analysis was performed using GraphPad Prism Version 6.0 (San Diego, CA, USA).

## Results

To confirm that all tested compounds induced apoptosis in MDA-MB-231 cells, several biochemical tests were done such as flow cytometric analysis after annexin V-FITC and PI staining, MMP, DNA fragmentation, and concentration of pro-apoptotic factors.

**Fig. 1:**
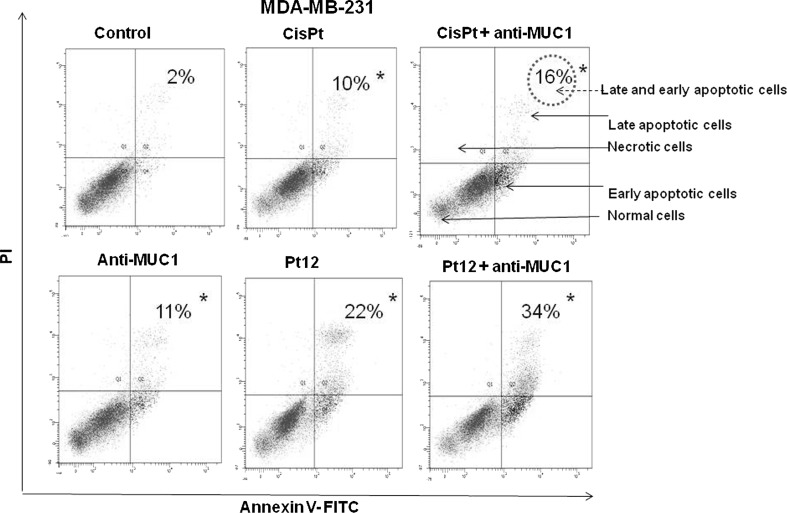
Flow cytometric analysis of breast cancer MDA-MB-231 cells after incubation with anti-MUC1 (10 μg/mL), Pt12 (10 μM), Pt12 + anti-MUC1 (10 μM + 10 μg/mL), cisplatin (10 μM), cisplatin + anti-MUC1 (10 μM + 10 μg/mL) for 24 h and staining with Annexin V and propidium iodide (PI). Dots with Annexin V−/PI−(Q3), Annexin V+/PI−(Q4), and Annexin V+/PI+(Q2) features represent intact, early apoptotic, and necrotic cells, respectively. *p < 0.05 versus control group

**Fig. 2:**
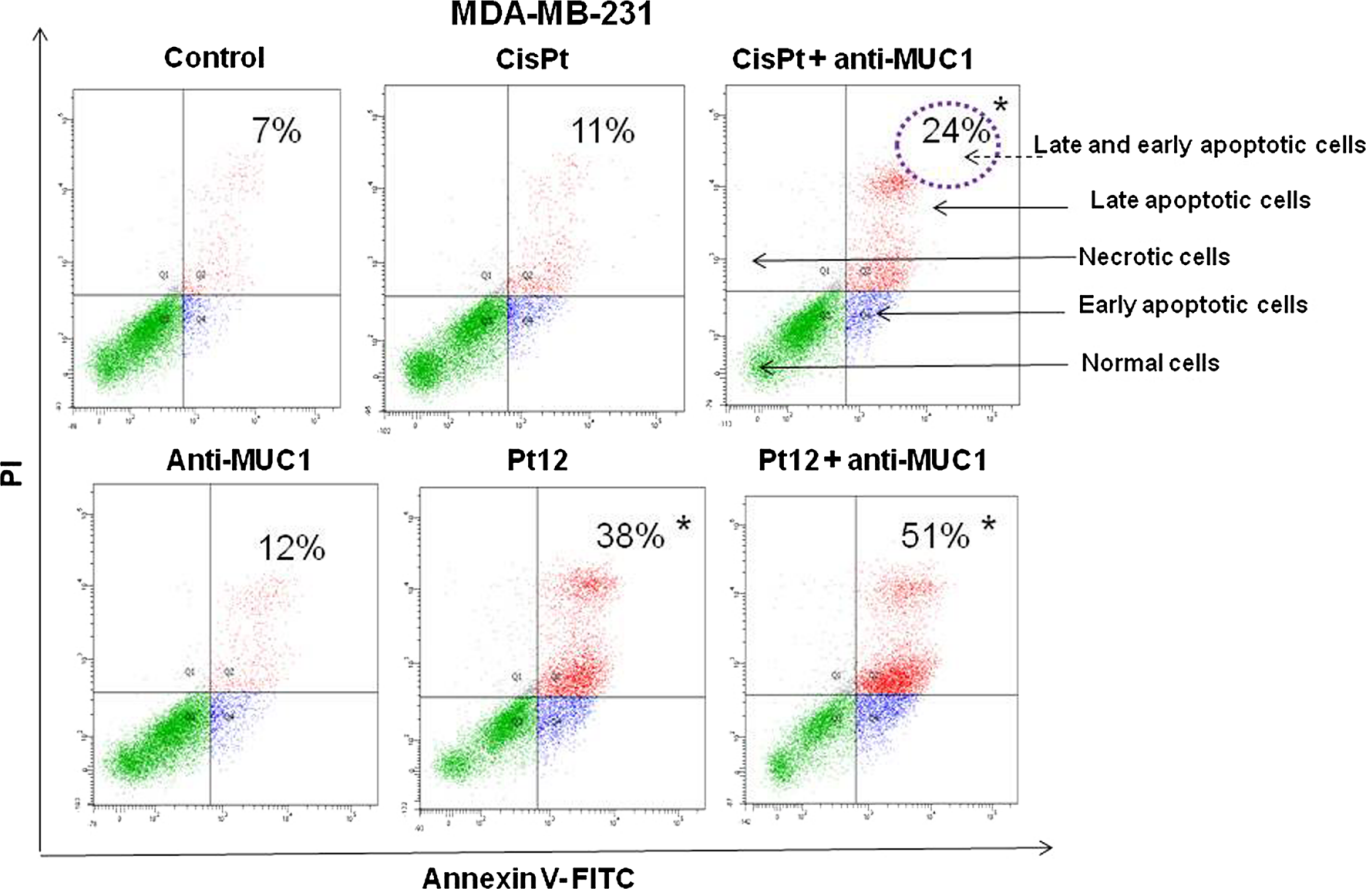
Flow cytometric analysis of breast cancer MDA-MB-231 cells after incubation with anti-MUC1 (10 μg/mL), Pt12 (10 μM), Pt12 + anti-MUC1 (10 μM + 10 μg/mL), cisplatin (10 μM), cisplatin + anti-MUC1 (10 μM + 10 μg/mL) for 48 h and staining with Annexin V and propidium iodide (PI). Dots with Annexin V−/PI−(Q3), Annexin V+/PI−(Q4), and Annexin V+/PI+(Q2) features represent intact, early apoptotic, and necrotic cells, respectively. *p < 0.05 versus control group

Flow cytometry analysis allowed to detect apoptotic cells (Annexin V+/PI−), life cells (Annexin V−/PI−), and necrotic cells (Annexin V+/PI+) after 24 and 48 h of incubation with drugs used in monotherapy (anti-MUC1, cisplatin, Pt12) and in combination (cisplatin + anti-MUC1, Pt12 + anti-MUC1) (Figs. 1, 2). The highest number of early and late apoptotic cells was observed after 24 h of incubation with Pt12 and anti-MUC1 (34 %). The value was higher compared to anti-MUC1 (11 %), cisplatin (10 %), Pt12 (22 %), and cisplatin with anti-MUC1 (16 %). All values were significantly increased (*p* < 0.05) compared to control samples, in which the number of early and late apoptotic cells reached 2 % (Fig. 1). After 48 h the apoptotic effect increased significantly, especially after combined treatment. We noticed that 51 % cells were in early and late apoptosis after incubation with Pt12 and anti-MUC1. The effect was stronger in comparison with monotherapy anti-MUC1 (12 %), cisplatin (11 %), Pt12 (38 %), and cisplatin used in combination with anti-MUC1 (24 %). In control cells, we detected 7 % of apoptotic cells (Fig. 2).

**Fig. 3:**
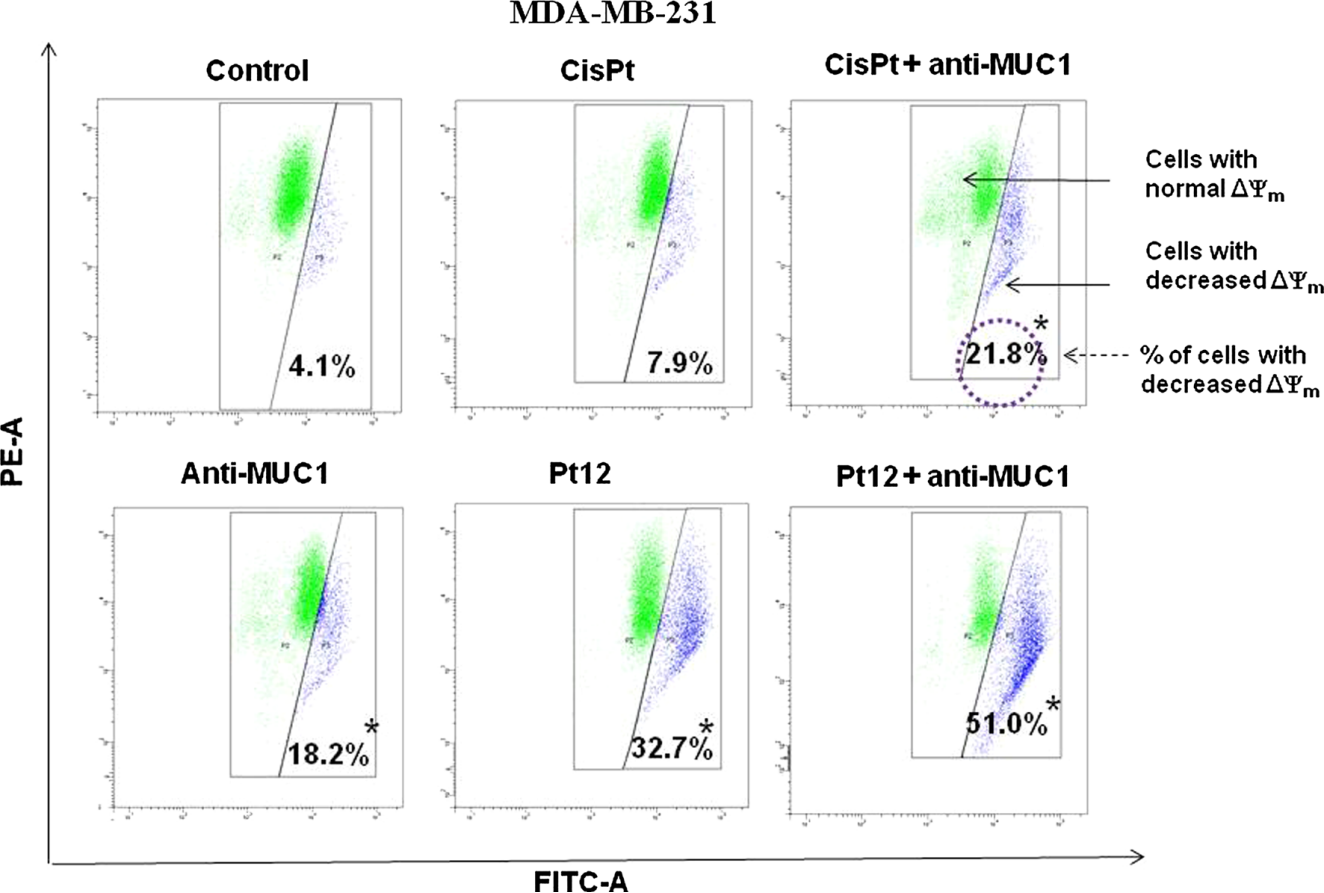
The loss of mitochondrial membrane potential of breast cancer MDA-MB-231 cells after 24 h incubation with anti-MUC1 (10 μg/mL), Pt12 (10 μM), Pt12 + anti-MUC1 (10 μM + 10 μg/mL), cisplatin (10 μM), cisplatin + anti-MUC1 (10 μM + 10 μg/mL) as measured JC-1 fluorescence. X- and Y-axes are *green* and *red* fluorescence, respectively. *p < 0.05 versus control group. (Color figure online)

**Fig. 4:**
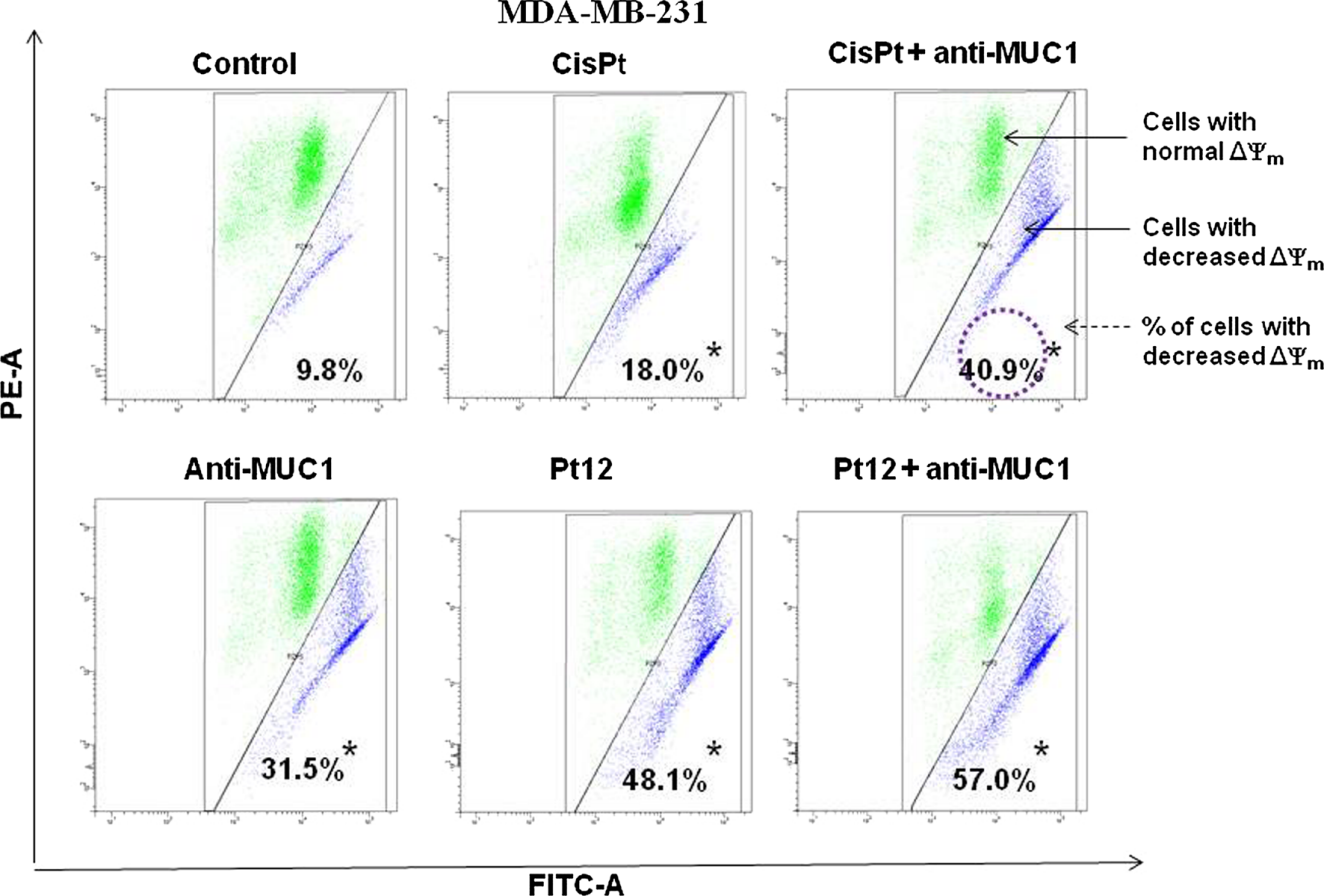
The loss of mitochondrial membrane potential of breast cancer MDA-MB-231 cells after 48 h incubation with anti-MUC1 (10 μg/mL), Pt12 (10 μM), Pt12 + anti-MUC1 (10 μM + 10 μg/mL), cisplatin (10 μM), cisplatin + anti-MUC1 (10 μM + 10 μg/mL) as measured JC-1 fluorescence. X- and Y-axes are *green* and *red* fluorescence, respectively. *p < 0.05 versus control group. (Color figure online)

Loss of MMP (ΔΨ_m_) has been shown to be an early event during apoptosis. To determine ΔΨ_m_, we used the membrane potential-sensitive probe JC-1, which forms JC-1 aggregates (with red color) at higher potential and JC-1 monomers (with green color) at low membrane potential, and the ratio between the red and green signals is a measure of ΔΨ_m_. The reduction of MMP is also associated with appearance of cytochrome c in cytosol [24]. Pt12 with anti-MUC1 reduced MMP in the highest rate. We observed that 51 % of cells had reduced ΔΨ_m_, whereas control cells only 4.1 %. The effect of reduction was weaker after 24 h of incubation with agents used in monotherapy. Anti-MUC1, cisplatin, Pt12 decreased the ΔΨ_m_ in breast cancer cells to 18.2, 7.9, and 32.7 %. We observed that 21.8 % cells had reduced ΔΨ_m_ after incubation with cisplatin used together with anti-MUC1 (Fig. 3). The stronger decrease was noticed after 48 h of incubation with drugs. Pt12 and anti-MUC1 caused the reduction of MMP in 57 % of breast cancer cells. The weaker effect in reduction of ΔΨ_m_ was observed after monotherapy with anti-MUC1 (31.5 %), cisplatin (18 %) and Pt12 (48.1 %). Cisplatin used with anti-MUC1 decreased the MMP in 40.9 % cells (Fig. 4).

**Fig. 5:**
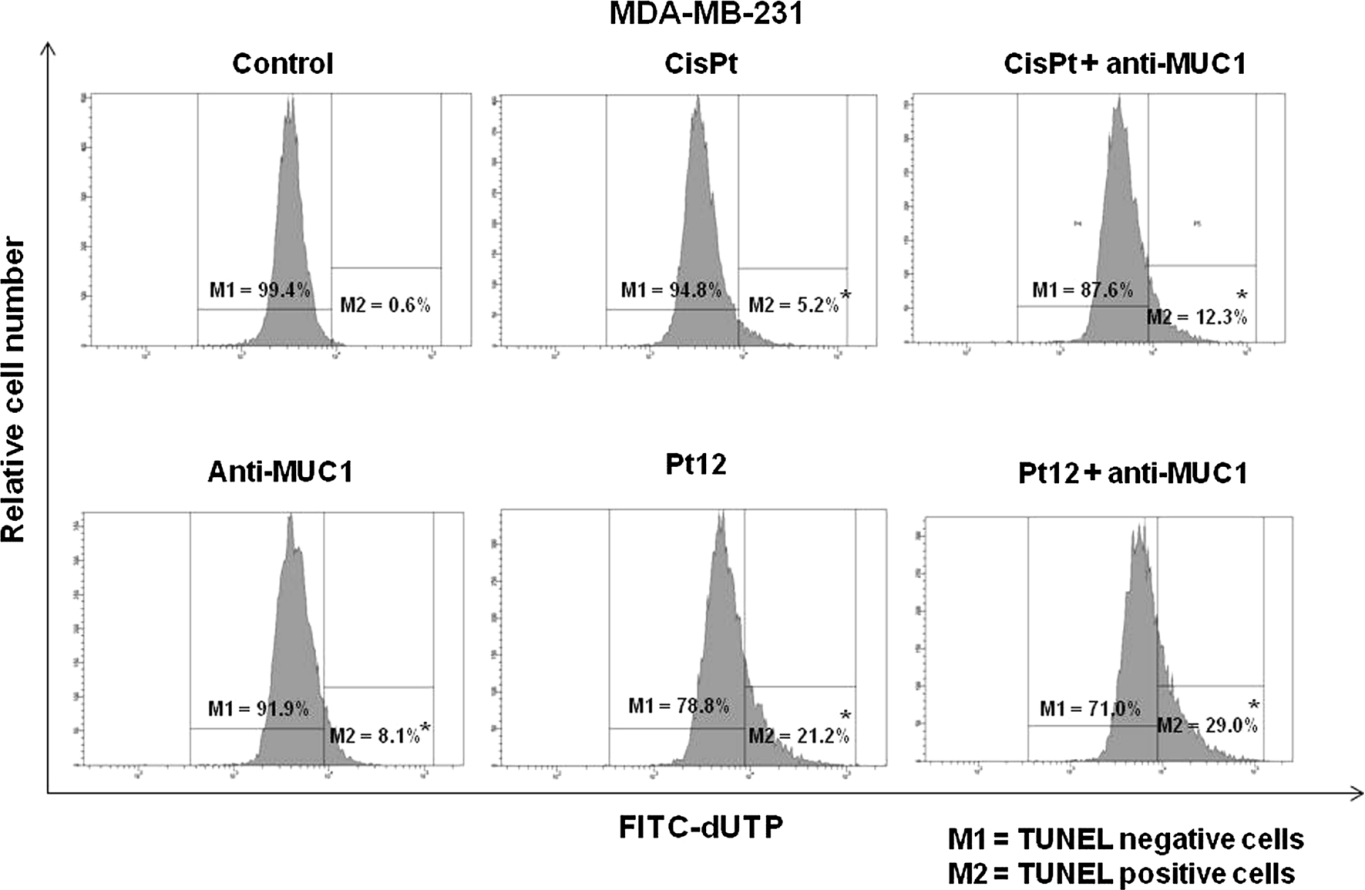
The DNA fragmentation of breast cancer MDA-MB-231 cells after 24 h incubation with anti-MUC1 (10 μg/mL), Pt12 (10 μM), Pt12 + anti-MUC1 (10 μM + 10 μg/mL), cisplatin (10 μM), cisplatin + anti-MUC1 (10 μM + 10 μg/mL) as presented  % of TUNEL positive and negative cells. *p < 0.05 versus control group

**Fig. 6:**
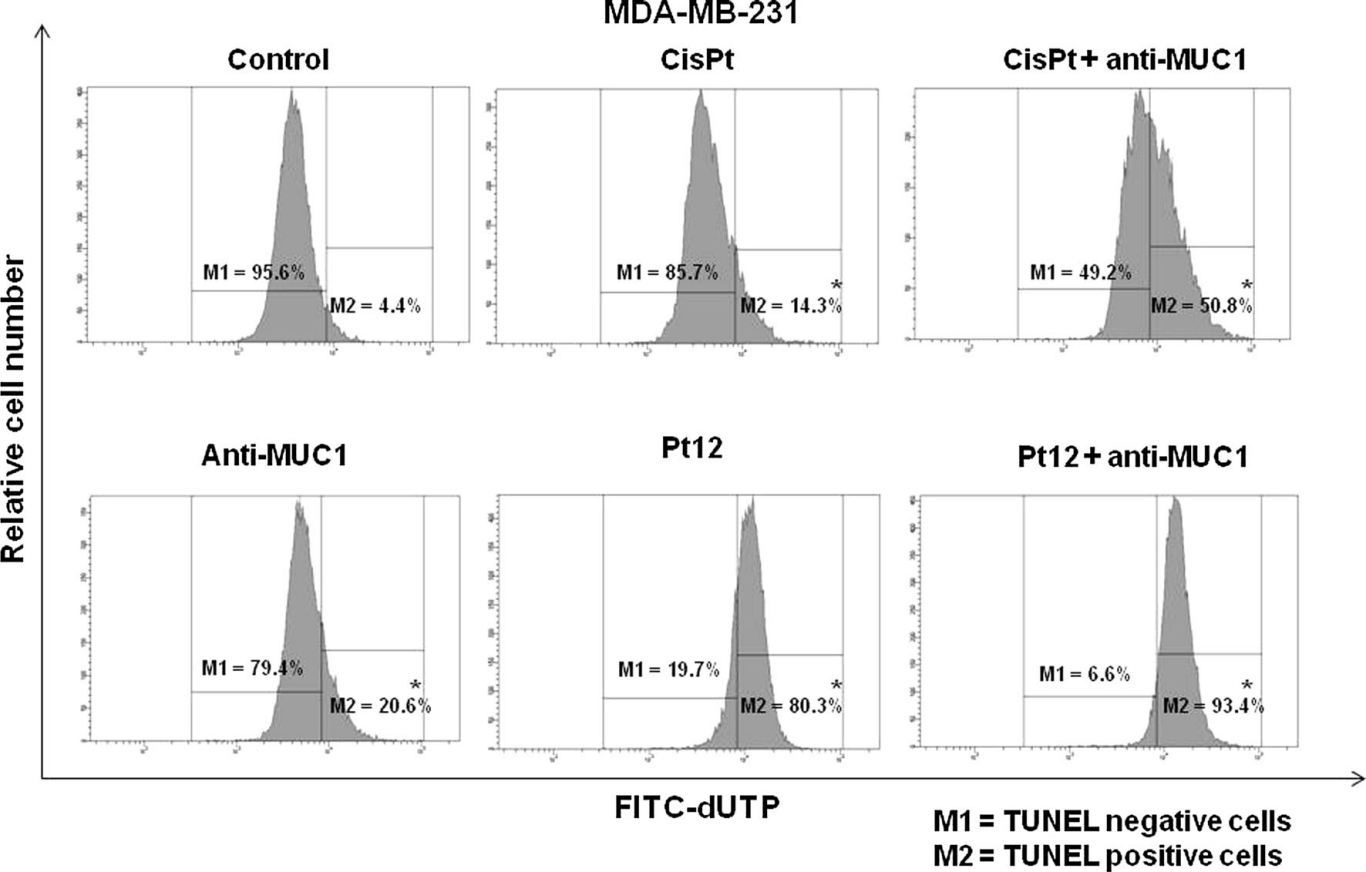
The DNA fragmentation of breast cancer MDA-MB-231 cells after 48 h incubation with anti-MUC1 (10 μg/mL), Pt12 (10 μM), Pt12 + anti-MUC1 (10 μM + 10 μg/mL), cisplatin (10 μM), cisplatin + anti-MUC1 (10 μM + 10 μg/mL) as presented  % of TUNEL positive and negative cells. *p < 0.05 versus control group

DNA degradation is one of the most characteristic moments of programmed cell death. It was performed using TUNEL assay. We observed TUNEL positive cells (M2) with fragmented DNA. Cells with double stranded DNA were called as TUNEL negative cells (M1). We noticed that 29 % of cells were TUNEL positive after 24 h of incubation with Pt12 and anti-MUC1. The DNA fragmentation was stronger than monotherapy by anti-MUC1 (8.1 %), cisplatin (5.2 %), Pt12 (21.2 %), and cisplatin used together with anti-MUC1 (12.3 %). Only 0.6 % of untreated cells had fragmented DNA (Fig. 5). After next 24 h of incubation, we observed that process of DNA fragmentation was stronger, especially after Pt12 (10 µM) with anti-MUC1 (10 μg/mL). We noticed that 93.4 % of cells had degraded DNA. We proved that Pt12 with anti-MUC1 were found to be more effective in degradation of DNA compared to anti-MUC1, cisplatin, Pt12, and cisplatin used in combination with anti-MUC1 (Fig. 6).

**Fig. 7:**
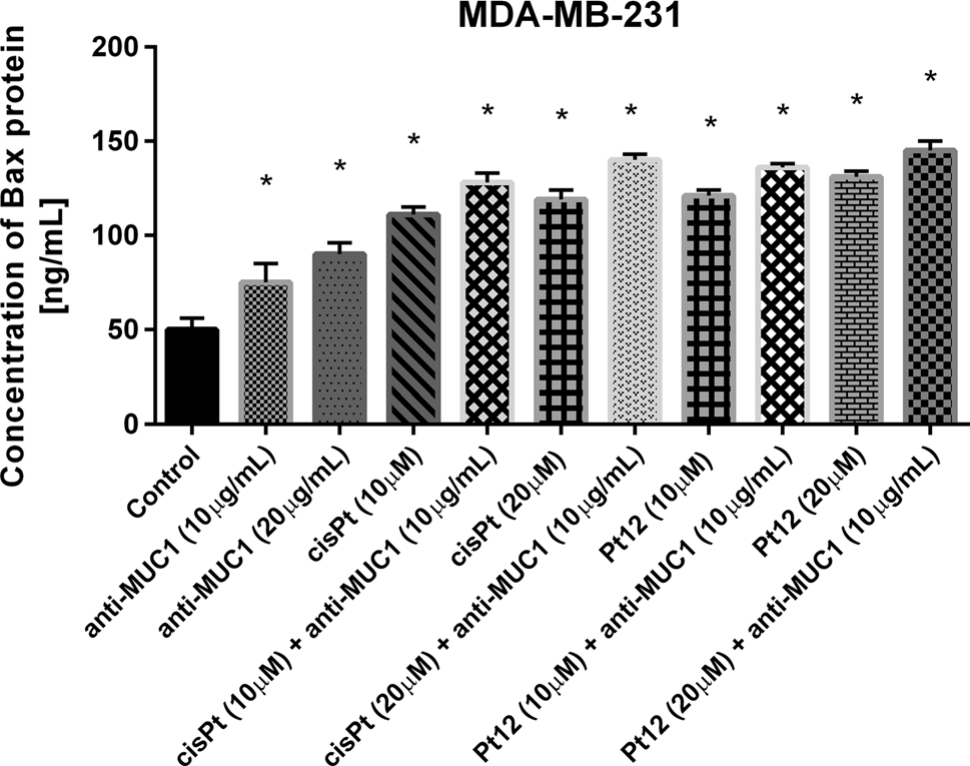
The concentration of pro-apoptotic Bax protein in breast cancer MDA-MB-231 cells after 24 h incubation with anti-MUC1 (10, 20 μg/mL), Pt12 (10, 20 μM), Pt12 + anti-MUC1 (10 μM + 10 μg/mL, 20 μM + 10 μg/mL), cisplatin (10, 20 μM), cisplatin + anti-MUC1 (10 μM + 10 μg/mL, 20 μM + 10 μg/mL). Data presented in ng/mL. *p < 0.05 versus control group

We measured the concentration of pro-apoptotic Bax protein (Fig. 7), which is necessary to release the cytochrome c from mitochondria to cytosol and improves mitochondrial pathway of apoptosis. All examined compounds significantly increased Bax concentration compared to untreated cells. After 24 h of incubation with agents used in monotherapy, the stronger inducer of Bax releasement was Pt12 in dose 20 µM. The effect was stronger than that produced by cisplatin and anti-MUC1 used alone. The highest increase in pro-apoptotic Bax protein concentration was observed after combined treatment of Pt12 (20 µM) together with anti-MUC1 (10 μg/mL). The concentration of Bax was 145 ng/mL compared to reference compound cisplatin used with anti-MUC1 in the same doses, where the level of Bax was: 140 ng/mL. The increase was almost 3 times stronger in comparison to control, where cells were untreated.

**Fig. 8:**
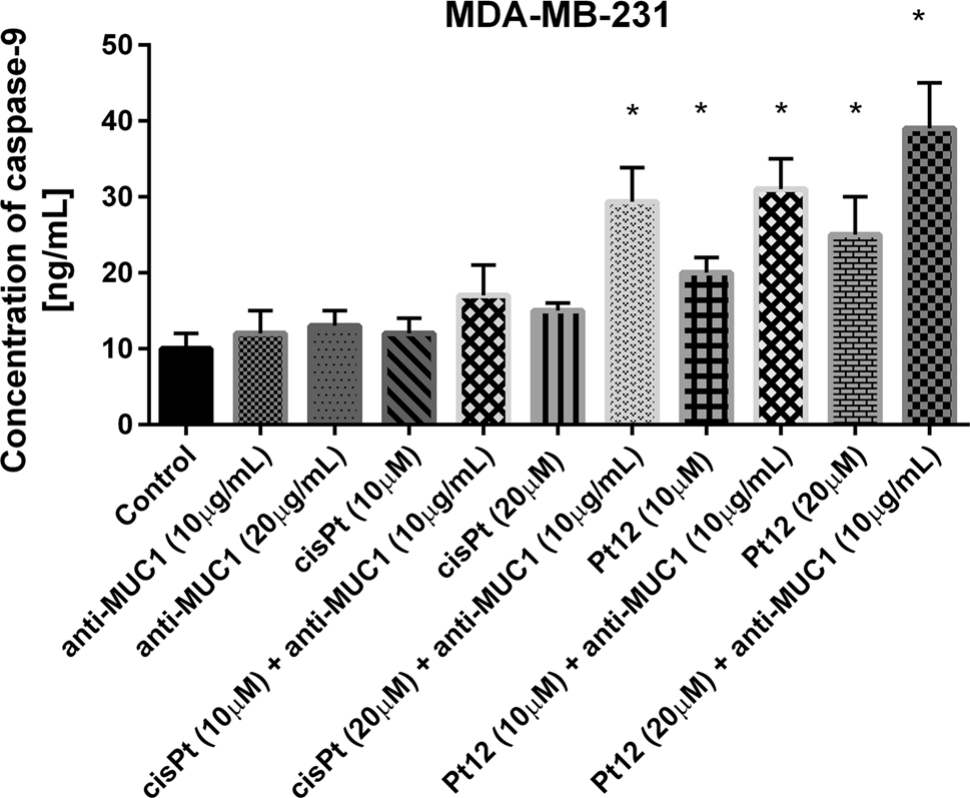
The concentration of caspase-9 in breast cancer MDA-MB-231 cells after 24 h incubation with anti-MUC1 (10, 20 μg/mL), Pt12 (10, 20 μM), Pt12 + anti-MUC1 (10 μM + 10 μg/mL, 20 μM + 10 μg/mL), cisplatin (10, 20 μM), cisplatin + anti-MUC1 (10 μM + 10 μg/mL, 20 μM + 10 μg/mL). Data presented in ng/mL. *p < 0.05 versus control group

Caspase-9 is a key component of mitochondrial pathway of apoptosis. We determined the concentration of caspase-9 after monotherapy and combination treatment. We observed that Pt12 was a stronger inducer of caspase-9 releasement compared to cisplatin and anti-MUC1 after 24 h of incubation with drugs. The strongest effect on caspase-9 releasement was determined after combined treatment with Pt12 (20 μM) and anti-MUC1 (10 μg/mL). The concentration of caspase-9 was 39 ng/mL. After cisplatin (20 μM) and anti-MUC1 (10 μg/mL) the level of caspase-9 was 29 ng/mL (Fig. 8). Significant increase in caspase-9 concentration was observed after combined treatment with chemotherapeutic agents used in dose 20 µM and anti-MUC1 in dose 10 μg/mL (*p* < 0.05).

**Fig. 9:**
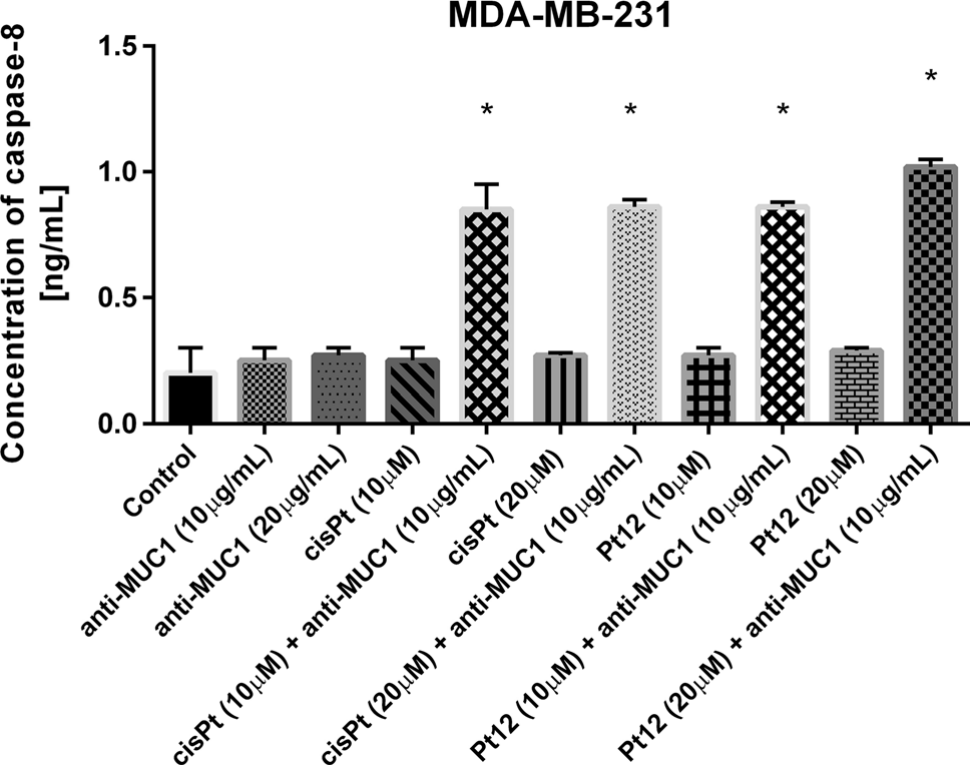
The concentration of caspase-8 in breast cancer MDA-MB-231 cells after 24 h incubation with anti-MUC1 (10, 20 μg/mL), Pt12 (10, 20 μM), Pt12 + anti-MUC1 (10 μM + 10 μg/mL, 20 μM + 10 μg/mL), cisplatin (10, 20 μM), cisplatin + anti-MUC1 (10 μM + 10 μg/mL, 20 μM + 10 μg/mL). Data presented in ng/mL. *p < 0.05 versus control group

We checked the effect of agents on apoptosis associated with extrinsic pathway. Caspase-8 is a main mediator of extrinsic pathway. All compounds increased the concentration of caspase-8 compared to control value, but the highest statistically significant increase was observed after Pt12 (20 μM) and anti-MUC1 (10 μg/mL), in comparison with cisplatin (20 μM) and anti-MUC1 (10 μg/mL), where the concentrations were 1.02 and 0.86 ng/mL, respectively. The effect was five times stronger compared with control cells (Fig. 9).

**Fig. 10:**
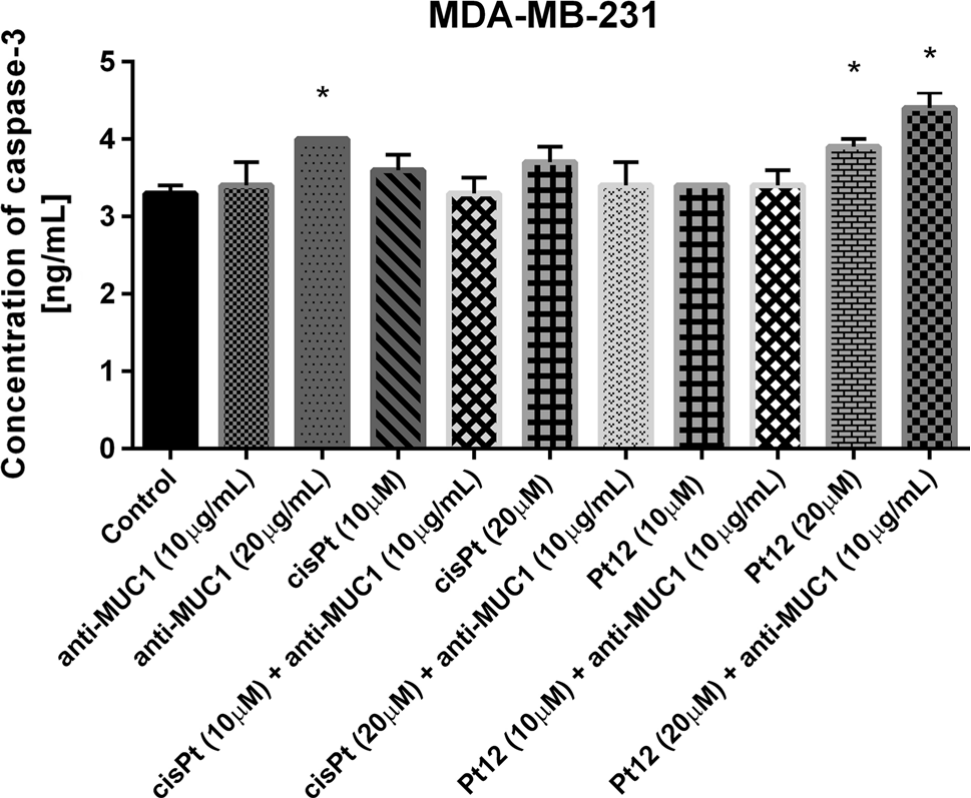
The concentration of caspase-3 in breast cancer MDA-MB-231 cells after 24 h incubation with anti-MUC1 (10, 20 μg/mL), Pt12 (10, 20 μM), Pt12 + anti-MUC1 (10 μM + 10 μg/mL, 20 μM + 10 μg/mL), cisplatin (10, 20 μM), cisplatin + anti-MUC1 (10 μM + 10 μg/mL, 20 μM + 10 μg/mL). Data presented in ng/mL. *p < 0.05 versus control group

Finally, we checked the concentration of caspase-3 in cell lysates after 24 h of incubation with drugs used in monotherapy and combination therapy (Fig. 10). The concentration after Pt12 (20 μM), cisplatin (20 μM) was 3.9 versus 3.7 ng/mL. The strongest statistically significant effect was observed after Pt12 (20 μM) with anti-MUC1 (4.4 ng/mL). Our results proved that combination therapy is more effective strategy in increasing the levels of pro-apoptotic markers compared to monotherapy.

## Discussion

Our preliminary studies showed that Pt_2_(4-ethylpyridine)_4_(berenil)_2_ (Pt12) used together with anti-MUC1 antibody is more cytotoxic than Pt12 alone or cisplatin with anti-MUC1 in breast cancer MCF-7 and MDA-MB-231 cells. The anti-proliferative effect of Pt12 with anti-MUC1 was dependent on the estrogen receptor status of the breast cancer cells [20]. Pt12 with anti-MUC1 was proved to be a stronger inhibitor of collagen biosynthesis in breast cancer cells compared to Pt12 alone and cisplatin with anti-MUC1. Understanding the pathways by which Pt12 with anti-MUC1 induces cell death can provide information necessary to target-specific cell death pathways in the treatment of breast cancer. In our study, the induction of apoptosis by new platinum complex with anti-MUC1 in human MDA-MB-231 breast cancer cells was confirmed by several biochemical markers, such as phosphatidylserine externalization, loss of MMP ΔΨ_m_, DNA degradation, Bax, and caspase-3, -8, -9 levels. All compounds induced apoptosis of breast cancer cells via mechanisms dependent on caspases activation and associated with MMP disruption, but the strongest effect was observed after Pt12 used in combination with anti-MUC1. In the literature appeared some data about monoclonal antibodies against MUC1. The positive results were obtained after the application of anti-MUC1: C595, GP1.4, HMFG2, ^90^Y-muHMFG1, and small molecule inhibitors GO-203 and PMIP [4]. The antibody C595 is a murine antibody and it represents the subclass of IgG3. C595 antibody and docetaxel strongly induces apoptosis in ovarian cancer cells in vitro, and in vivo inhibits tumor growth [25, 26]. GP1.4 antibody inhibits proliferation and invasion of tumor cells. HMFG2 antibody also reduces tumor burden in a mouse model of pancreatic cancer. Studies on ^90^Y-muHMFG1 antibody that recognizes glycosylated extracellular MUC1 domain show the long-term survival of people. Some antibodies successfully completed preclinical phases. An example is a HMFG1 antibody, which is currently in the third phase of the clinical trial. MUC1 vaccine containing the cDNA (M-FP) is also tested in the third phase of clinical trial in patients with breast cancer [4]. The most promising is combining monoclonal antibodies with chemotherapeutic agents. Some studies on the use of other monoclonal antibodies (trastuzumab, pertuzumab) and carboplatin in the treatment of breast cancer is currently in phase II of clinical trials called TRYPHAENA. In clinical trials, there is also a 106 amino acid peptide against MUC1 used in conjunction with the peptide HER-2/neu, and GM-CSF. Moreover, Stimuvax which contains 25 amino acid sequences and recognizes the protein core of MUC1 is tested in the third phase of clinical trials [27]. All these findings have provided the experimental basis for targeting MUC1 in patients with diverse carcinomas (breast, prostate, lung cancer, and others with epithelial origin) that express this oncoprotein. According to the literature MUC1-C protects against the apoptotic response to treatment with diverse chemotherapeutic agents [9, 28–30]. The anti-apoptotic effect might be associated with interaction between MUC1-C and Bax protein. It leads to blockade of Bax dimerization and releasement of cytochrome c. Our results proved the highest levels of Bax protein after Pt12 and anti-MUC1 treatment compared with control. Uschida et al. reported that inhibition of the MUC1-C oncoprotein is synergistic with cytotoxic agents in the treatment of breast cancer. They proved that combining MUC1-C inhibitor (GO-203) with taxol or doxorubicin induced late apoptosis/necrosis and activated caspase-3 and caspase-7 in ZR-75-1 cells [31]. Our research proved that combining monoclonal antibody with potential chemotherapeutic agent resulted in better pro-apoptotic activity. Pt12 with anti-MUC1 activate both: extrinsic and intrinsic pathways of programmed cell death, especially strongly induced extrinsic pathway associated with caspase-8. The concentration of caspase-8 was a few times stronger compared to monotherapy. Monoclonal antibody against MUC1 increased sensitivity of breast cancer cells to the platinum(II) compound. Taken together, combining antibodies with drugs is more effective strategy in treatment of carcinoma with high expression of MUC1.
